# A probabilistic approach to lifetime design of offshore platforms

**DOI:** 10.1038/s41598-023-34362-x

**Published:** 2023-05-02

**Authors:** Mehdi Hajinezhadian, Behrouz Behnam

**Affiliations:** School of Civil and Environmental Engineering, Amirkabir University of Technology, 424 Hafez Street, Tehran, Iran

**Keywords:** Natural hazards, Ocean sciences, Engineering

## Abstract

Offshore platforms are considered critical infrastructure as any disruption in their lifetime service can rapidly result in a great loss to arise. While these structures are often designed for their initial construction cost, it is worth considering a lifetime-based design so that both direct and indirect costs are involved in the design process. Here, a probabilistic-based approach to life-cycle-cost (LCC) analysis of offshore platforms is proposed. A fixed offshore platform is designed first based on the current design regulations and for a 100-year return period. For the effect of LCC on design optimization, the simultaneous effect of the wave, current, and wind merging are probabilistically considered. The structural elements are designed for five different models; one model based on the current design requirements and the rest for more than the requirements. The LCC of each model is accordingly determined. The results show that the code-based model is not optimal when is compared with a lifetime cost period; it is necessary to increase the size of structural elements by up to 10% to meet an optimum point. Results show that with a 5% increase in the initial cost, a decrease in the LCC up to about 46% is observed. The work presented here is to stimulate stakeholders to promote the LCC-based design of important structures to reduce lifetime costs.

## Introduction

Offshore platforms are established to extract oil and gas reserves from the depths of the seas. Due to their importance, in case of any interruption in their routine activities, their stakeholders can sustain huge losses^1,2^. While offshore platforms are normally designed based on available standards, recent experiences- such as the one occurred in the Gulf of Mexico and caused extensive damage- have revealed that a design based on the current regulations is not necessarily economically optimal^3,4^. The common belief in the optimal structural design is that it should decrease the initial construction cost; however, the lifetime cost may be much more than the initial cost- in the current regulations, this important point is not addressed. The lifetime cost is termed as life-cycle cost (LCC) and is divided into primary and secondary costs. The primary cost includes purchasing materials, wages, construction, design, implementation, transportation, setup, and the platform test^5^; the secondary cost relates to the cost of the operation period and the lifetime risks to the structure. The damage can be defined in form of losing a platform and investment opportunities, injuries and casualties of staff, cost of stopping the oil and gas extraction, re-testing and -starting the platform, equipment loss, and repair and retrofitting.

The LCC-based design for conventional structures with a probabilistic approach has enjoyed some attention over the last decades, e.g. Liu and Neghabat^6^, Asiedu and Gu^7^, Lagaros et al.^8^, Uddin and Mousa^9^, Marzouk et al.^10^, Behnam^11^, Hassani et al.^12^, Talaslioglu^13–15^, and Jebelli et al.^16^. As for offshore platforms, overall, rare studies have yet employed the above-noted approach but there are studies to probabilistically model the loads' characteristics, or to account for indirect costs, particularly environmental ones. Some studies have estimated wind speed and wave height with likelihood models. Heredia-Zavoni et al.^17^ determined the failure probability of steel jacket platforms under fatigue damage defining limit state functions for their applied loads. Lee et al.^18^ estimated extreme wind speed using Gumbel and Weibull distributions. To study waves directly, Kwon et al.^19^ employed a statistical method for the estimation of extreme sea levels. Bea et al.^20^ generalized the life-cycle risk characteristics of offshore platforms based on reliability and risk assessment, considering internal, and external factors. Pinna et al.^21^ determined the optimum design of monopod platforms by cost-effective criteria and considered the economic consequences of failure and the proportion of the construction cost. Leon and Alfredo^22^ proposed a reliability-based cost–benefit optimal decision model for the risk management of oil platforms considering the integration of social and economic issues into a management decision framework and formulated the cost functions as functions of the damage levels. Ang and Leon^23^ analyzed the offshore structures constructed in the Mexico Bay with the cost functions as damage index and applied it to an optimum design method. Hasofer^24^ modeled the definition of reliability for structural elements. Rockweiss and Flessler^25^ proposed a numerical method for calculating structural reliability. The wavelength analysis method, which is based on the new bound wave theory, was introduced by Zeinoddini et al.^26^ as the reliability theory. Ricky et al.^27^ examined two fixed marine jackets for possible failure; they estimated the probability of failure in different directions. The level of failure was divided into three categories based on the reliability index: mild, moderate, and severe. Lee et al.^28^ designed a marine structure and calculated the probability of failure for different return periods and their corresponding estimated indirect costs. Then, determining the minimum LCC of a target function, they designed the structure for optimal loads. Guédé^29^ introduced a method for risk-based assessment and developed an inspection plan as part of a structural integrity management plan for fixed offshore platforms. Ayotunde et al.^30^ assessed the correctness of high-power energy storage technologies for offshore platforms from an LCC point of view. Vaezi et al.^31^ investigated first the effects of a specific structural system on the dynamic response of offshore platforms and then proposed an optimization framework to be employed in the design of marine structures under applied loads. Qi et al.^32^ developed a time-dependent corrosion model for mobile offshore platforms. Li and Wang^33^ proposed an approach to compute the environmental benefits of optimized offshore platforms. Katanyoowongchareon et al.^34^ performed a reliability analysis and quantitative risk assessment to optimize the direct cost of offshore platforms. Colaleo et al.^35^ assessed the environmental and economic impacts of an existing offshore platform from a LCC point of view. Janjua and Khan^36^ developed an eco-efficiency framework for environmental and economic impact assessment of offshore platforms. Heo et al.^37^ developed an optimization framework for an offshore energy transition to assess the fatigue damage.

While more studies regarding offshore platforms can be reviewed, it is understood that advancing a proper approach to understanding the optimal economic design over the lifetime of structures is still of paramount importance; this can effectively be addressed through a reliability-based approach in which it can minimize the LCCs of offshore platforms. Here, it is controlled whether the design of offshore platforms under the requirements of the American Petroleum Institute (API), as a widely employed regulation, is optimum if a probabilistic-based approach to lifetime design is taken into account. Here, the probability of failure (PoF) and its consequences during the lifetime use of offshore platforms are determined, and an LCC-based design approach is proposed. Information about environmental conditions such as the maximum wave height, maximum wind speed, and the velocity of the sea current is estimated with functions such as Gumbel and Weibull distributions. To do that, an offshore platform is first designed as per API-RP2A^38^ with a 100-year return period; completely random dynamic loads of the time history are then analyzed where they are introduced as random variables for each structural element. Suitable distributions are obtained using Easy-Fit software^39^ for the values of random variables. Using MATLAB and based on the first-order reliability method (FORM), programmed reliability and the reliability index are calculated followed by the PoF of each element^24^. The LCC of the designed platform is next calculated; by changing the dimensions of the structural elements, the results are modified until the LCC is minimized. The work here uses time history dynamic analysis to increase the accuracy of the results. The damage index is determined based on the FORM and under two performance functions of tension and compression where their minimum values are used as the critical reliability index. As well, the critical direction is determined based on a probabilistic-based approach.

The proposed approach in the current study can determine the minimum lifetime costs of offshore platforms as a function of failure probability. Minimizing such costs allows for deriving an optimum design criterion. With potential consequences varying from a local buckling to significant deformation, there are no recommended criteria yet that demarcate when to repair or to account for annual damage in an element. In this context, a reliability index-based repair criterion can highly address the above concerns. It is worth noting that structural systems are often designed with the aim that the structural components will be under their threshold stress/strain values if subjected to design loads. Nevertheless, this does not mean that if, for instance, the stress/strain value in a structural component goes over the threshold, the total structural failure, i.e. progressive collapse, will occur. Investigating the progressive collapse resistance of structural systems is an important research subject, which has received much attention so far, but it is not within the scope of the study here.

## Methodology

**Figure 1:**
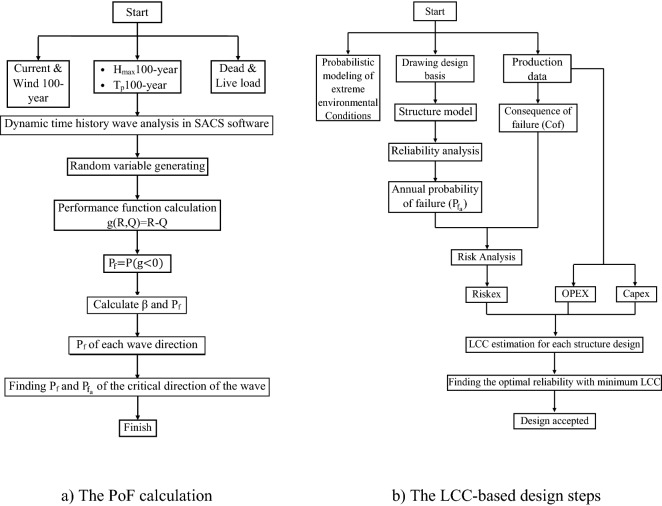
Steps of determining the PoF and LCC of offshore platforms.

To design an offshore platform, the environmental loads and the probability of their occurrence are first introduced, and analytical methods to find PoF and LCC models are then presented. The PoF and the process of an LCC-based design are presented in Fig. 1a,b as discussed in the following sections.

### Environmental conditions

Offshore platforms are exposed to various forces, including wind, sea currents, and waves, which might be uniform or a function of time. Sea conditions during stormy weather are also considered. The forces created by wind are often uniform and act only on the upper part of the platform from the sea surface. Sea currents impose uniform flow to the underwater parts of the structure^40^. Waves are often the largest dynamic and dominant environmental forces on fixed offshore platforms^41^. Due to the oscillating nature and randomness of the wave height and their uncertainty, a criterion called reliability index is used to obtain the PoF of elements^42^. Predicting the maximum annual wave height is one of the basic parameters in designing for all eight main and sub-geographical directions. Equation 1 gives the general form of Weibull's three-parameter distribution function to predict the maximum annual wave height on the offshore platform^43^:

1 $${f}_{{H}_{s}}\left(h\right)=\frac{{\beta }_{{H}_{s}}}{{\alpha }_{{H}_{s}}}{\left(\frac{h-{\gamma }_{{H}_{s}}}{{\alpha }_{{H}_{s}}}\right)}^{{\beta }_{{H}_{s}}-1}exp\left\{-{\left(\frac{h-{\gamma }_{{H}_{s}}}{{\alpha }_{{H}_{s}}}\right)}^{{\beta }_{{H}_{s}}}\right\}$$

where *f*_*Hs*_ (ℎ) is the long-term probability density function of the specified height of the wave, *α*_*Hs*_ is the scale parameter, *β*_*HS*_ is the shape parameter, and *γ*_*HS*_ is the position parameter. Here, climatological data are obtained from the Glenn Report^43^. The data are then fitted with the maximum likelihood estimation method by a three-parameter Weibull probability density function to obtain the wave hazard curve. This distribution for a certain direction is obtained assuming that the wave comes only from an assumed direction during one year (see Table 1).

**Table 1:** Weibull's three-parameter distributions function for different directions in the Persian Gulf^43^.

Weibull parameter	North	North East	East	South East	South	South West	West	North West	All directions
${\gamma }_{{H}_{s}}$	0	− 0.169	− 0.08	− 0.135	− 0.09	− 0.15	− 0.03	− 0.04	− 0.106
${\beta }_{{H}_{s}}$	1.189	1.352	1.325	1.326	1.352	1.435	1.3	1.21	1.295
${\alpha }_{{H}_{s}}$	0.662	0.718	0.718	0.945	0.932	0.832	0.825	0.831	0.975

Wind speed varies based on altitude and wind time; therefore, to determine wind speed, the wind time and height of the base should be determined^40^. The base velocity is recorded at an altitude of + 10 m relative to the zero level of the sea and the average velocity per minute. When wave or wind data is not available, Eq. (2) can be used. The interaction between significant wave height and wind speed is estimated by the Gumbel distribution function for each return period^44^. As there is a correlation between wind speed and wave height, changes in wind speed can affect wave height.

2 $$ SWH = 0.0246*\left( {wind\;velocity} \right)^{2} $$

Currents are divided into two types: wind currents and tidal currents. It is assumed that the velocity of currents, which is affected by the wind at the sea level, is approximately 1% of the wind velocity^45^; their velocity linearly decreases to the seabed. Based on the information obtained from measurements on vessels near the site, the maximum and minimum flow velocities are estimated.

### Reliability analysis

The performance of a structure is generally considered to have been rejected when the load-bearing capacity of the structure is less than the nominal values of the applied load. Often, a system structure is made up of numerous components; the reliability method, therefore, provides the relationship between the reliability of a component and a system. Many uncertainties might be faced during the design process; unpredictable loads such as waves and wind loads, sea currents, elastic modulus, and yield stress, are examples of uncertainties^46^. Hence, resistance and load parameters are defined as random variables^42^. In general, the state of structural failure has different meanings; the boundary between good and bad performances of a structure can be the definition of failure. This boundary is expressed by the limit state functions^41^. If *R* is resistance, and Q is loading on the structural element, the performance function is defined via Eq. (3).

3 $$g\left(R,\mathrm{ Q}\right)=R-\mathrm{Q}$$

when the performance of a structure is unsatisfactory, the possibility of failure becomes significant and it is expressed by Eq. (4).

4 $${P}_{f}=P\left(R-\mathrm{Q }<0\right)=P\left(g<0\right)$$

For determining the reliability Index, the random variables should be converted to a dimensionless format. For two variables and the linear performance function, the reliability index β can be defined as the shortest distance from the origin to the failure line. If the boundary limit function has N random variables in standard space and is non-linear, it should be first linearized using the Taylor exponent; the reliability index is then calculated via FORM. In the Hasofer-and-Lind reliability index method, the linearization point of the limit state function in the Taylor expansion uses the design point instead of the mean point^24^. This design point is not clear at first and is achieved through a trial and error process. The linearization of the limit state function is approximated where all equivalent functions share the same point, that is, the point that holds in Eq. (5).

5 $$ g\left( {z_{1}^{*} ,z_{2}^{*} , \ldots ,z_{n}^{*} } \right) = 0 $$

where ${z}_{i}$ is the reduced variable in a standard or dimensionless format, and the notation ${z}_{\mathrm{i}}^{*}$ is used for the design points in the reduced coordinates. We start this process with the design point, which is z*. Using FORM, a partial derivative of the limit conditions concerning random variables around design points is first obtained as given in Eq. (6), and then a partial derivative matrix is formed.

6 $$ \left[ {\text{G}} \right] = \left[ {\begin{array}{*{20}c} {G_{1} } \\ \vdots \\ {G_{n} } \\ \end{array} } \right];\;{\text{G}}_{{\text{i}}} = - \frac{{\partial_{g} }}{{\partial {\text{z}}_{{\text{i}}} }} $$

where G_1_ to G_n_ are partial derivative matrices related to N random variable. The first estimate of the reliability index is obtained using Eq. (7).

7 $$ {\upbeta } = \left[ {\text{G}} \right]^{T} \left[ {{\text{z}}^{*} } \right] \times \left( {\frac{1}{{\sqrt {\left[ {\text{G}} \right]^{T} \left[ G \right]} }}} \right);\; \left[ {z^{*} } \right] = \left[ {\begin{array}{*{20}c} {{\text{z}}_{1}^{*} } \\ \vdots \\ {{\text{z}}_{n}^{*} } \\ \end{array} } \right] $$

To determine the next design point, the sensitivity matrix α is calculated using Eq. (8).

8 $$ \left[ \alpha \right] = \left[ G \right] \times \left( {\frac{1}{{\sqrt {\left[ {\text{G}} \right]^{T} \left[ G \right]} }}} \right) $$

Then, the values of the new design points are obtained using Eq. (9).

9 $$ z_{i}^{*} = \beta \alpha_{i} $$

As these values are in the standard space, we take them to the main space as given in Eq. (10).

10 $$ x_{i}^{*} = z_{i}^{*} \sigma_{xi} + \mu_{xi} $$

where $\mu_{xi}$ is the value of the basic random variable and $\sigma_{xi}$ is the standard deviation of the basic random variable. The notation $x_{i}^{*}$ is used for the design point in the regular coordinates. Calculating this index and determining the basic values to achieve the desired convergence, the reliability method is repeated. In this research, the optimal convergence is considered to the extent that the difference between the reliability indices and the basic values of the design obtained from two replications is equal to or less than 0.001. After obtaining the convergence conditions, the reliability index *β* is achieved. The PoF corresponding to the *β* is calculated using Eq. (11).

11 $$ \beta = - \varphi^{ - 1} \left( {P_{f} } \right)\quad or\quad P_{f} = \varphi \left( { - \beta } \right) $$

In the reliability analysis of offshore platforms, it is relevant to take into account a time domain progressive deterioration because of damage accumulation. Here, the damage is due to the loading and unloading associated with operational wave loading. The accumulation of damage increases the risk of a potential failure during extreme events. In Eq. (12), $P_{{f_{a} }}$ is the annual probability of failure of the structure; it is the probability of lateral wave loading exceeding the ultimate resistance of the jacket in any given year. Assuming that a year is independent of another year, the cumulative probability of failure for *t* years can hence be calculated^17^.

12 $$ P_{f} \left( t \right) = 1 - \mathop \prod \limits_{{{\varvec{k}} = 1}}^{{\varvec{t}}} \left[ {1 - P_{{f_{a} }} \left( {\varvec{k}} \right)} \right] $$

where $P_{{f_{a} }} ({\varvec{k}})\user2{ }$ is the annual probability of failure in year *k*. Although maximum annual storm events can be considered independent, the accumulation of damage in the structure and its deterioration is not. Hence, the estimation of the $P_{f}$ in Eq. (12) is somehow conservative. Therefore, if M is the number of mutually exclusive damage states considered in the analysis, each damage state can be defined by a level of damage in a particular element or a set of elements. Then the $P_{{f_{a} }}$ in year t can be expressed via Eq. (13)^17^.

13 $$ P_{{f_{a} }} \left( t \right) = P_{{f_{a,0} }} P_{md} \left( {\varvec{t}} \right) + \mathop \sum \limits_{{{\varvec{i}} = 1}}^{{\varvec{M}}} P_{{f_{a,i} }} P_{di} \left( {\varvec{t}} \right) $$

where $P_{{f_{a,0} }}$ is the annual conditional probability of failure given that there is no damage, $P_{{f_{a,i} }}$ is the annual conditional probability of failure given the state of damage *i*, $P_{di} ({\varvec{t}})$ is the probability of occurrence of damage state *i*, in year *t*; and $P_{md} ({\varvec{t}})\user2{ }$ is the probability of no damage in the structure in year *t* as shown in Eq. (14).

14 $$ P_{md} \left( {\varvec{t}} \right) = \user2{ }\mathop \prod \limits_{{{\varvec{i}} = 1}}^{{\varvec{M}}} \left[ {1 - P_{di} \left( {\varvec{t}} \right)} \right] $$

Finally, the annual reliability index ($\beta_{a}$) throughout the design lifetime is calculated using Eq. (15)^17^. In Eqs. (11) and (15), $\varphi$ is the standard cumulative distribution function.

15 $$ \beta_{a} = \varphi^{ - 1} \left( {1 - P_{{f_{a} }} \left( {\varvec{t}} \right)} \right) $$

### LCC determination

As pointed out earlier, LCC has two parts; the initial costs and secondary costs. The initial costs of construction, operation, repair, and maintenance are all estimated^47^. These items should be included in the actual cost. In this research, a 25-years operation is considered as the lifetime. The damage cost caused by environmental loads is considered; the LCC is then calculated using Eq. (16).

16 $$ {\text{LCC}} = {\text{CAPEX}} + {\text{OPEX}} + {\text{RISKEX}} $$

where CAPEX, OPEX, and RISKEX are the initial direct cost, secondary cost over the operation period, and secondary cost due to the offshore platform risk, respectively. Equation 17 expresses CAPEX, which consists of manufacturing costs ($${C}_{m}$$), and the coefficient of manufacturing costs labeled as *a*. CAPEX can be estimated based on manufacturing cost that is calculated from the dimensions of the components of the platform. Equation 15 expresses the direct cost which consists of engineering design revisions and update costs ($${C}_{ed}$$), general administration and project control cost ($${C}_{ga}$$), contractor items equipment, and bulk material cost ($${C}_{cle}$$), general service, vendor assistance, and third-party services cost ($${C}_{gs}),$$ yard fabrication cost ($${C}_{yf}),$$ mobilization and demobilization cost ($${C}_{md}),$$ site work, and installation work cost ($${C}_{sw}),$$ and pre-commissioning and performance test cost ($${C}_{pt}).$$

17 $$ CAPEX = C_{m} + C_{md} + C_{sw} = C_{m} \times a $$

18 $$ C_{m} = C_{ed} + C_{ga} + C_{cle} + C_{gs} + C_{yf} + C_{pt} $$

Equation 19 expresses OPEX, which consists of the replacement of expendables cost ($${C}_{re}$$), corrosion checks, and paint cost ($${C}_{cp}),$$ machine repairs, technical inspection of equipment cost ($${C}_{rti}$$), repairing and maintaining petroleum wells cost ($${C}_{rpw}$$), helicopter cost ($${C}_{hl}),$$ and the cost of floating logistics ($${C}_{flo}$$). OPEX can be estimated based on the CAPEX.

19 $$ OPEX = C_{re} + C_{cp} + C_{rti} + C_{rpw} + C_{hl} + C_{flo} = b \times {\text{CAPEX }} \times LS \times P_{w} $$

In Eq. (16), the present worth factor (*Pw*) should be considered because OPEX and RISKEX represent the expected cost or loss during the lifespan of the structure. *LS* is the lifespan of the platform, and *b* is the coefficient of CAPEX for OPEX^48^. When the structural system is designed, the probability of structural failure is estimated based on the selected probabilistic model. RISKEX, which includes the economic loss from the damaged structure during its life is estimated from the annual PoF. The RISKEX is calculated through the multiplication of damage cost (${C}_{d}$), the annual probability of failure (${P}_{{f}_{a}}$), the $LS$, and the ${P}_{w}$ according to Eq. (20).

20 $$ RISKEX = \sum C_{d} \times P_{{f_{a} }} \times LS \times P_{w} $$

The annual damage cost is estimated via Eq. (21). The loss caused by the environmental loads is determined through the multiplication (${C}_{d}$) and (${P}_{{f}_{a}}$) for the four damage states^11^.

21 $$ \sum C_{d} P_{{f_{a} }} = C_{{d_{is} }} \times P_{{f_{a} \,is{ }}} + C_{{d_{m} }} \times P_{{f_{a} \,m{ }}} + C_{{d_{s} }} { } \times P_{{f_{a} \,s}} + C_{{d_{sv} }} \times P_{{f_{a} \,sv{ }}} $$

where ${C}_{{d}_{is}}$ is the damage cost for insignificant consequences*,*${C}_{{d}_{m}}$ is damage cost for minor consequences,${C}_{{d}_{s}}$ is the damage cost for significant consequences, ${C}_{{d}_{sv}}$ is the damage cost for severe consequences, ${{P}_{{f}_{a}}}_{is}$ is the annual probability of failure for insignificant consequences, ${{P}_{{f}_{a}}}_{m}$ is the annual probability of failure for minor consequences,${{P}_{{f}_{a}}}_{s}$ is the annual probability of failure for significant consequences, and ${{P}_{{f}_{a}}}_{sv}$ is the annual probability of failure with severe consequences. The relationships between structural performances and damage levels under dynamic loads during the 100-year return period are shown in Table 2^49^.

**Table 2:** Structural performance and damage levels under the dynamic loads.

Annual reliability index	Annual probability of failure	Damage state	Damage loss
3.72 $\le {\beta }_{a}$	${P}_{{f}_{a}}\le$ 0.0001	Insignificant	${C}_{R}$
3.09 $\le {\beta }_{a}$<3.72	0.001 $\ge {P}_{{f}_{a}}$>0.0001	Minor	${C}_{R}+{C}_{E}$
2.32 $\le {\beta }_{a}$<3.09	0.01 $\ge {P}_{{f}_{a}}$>0.001	Significant	${C}_{R}+{C}_{E}+{C}_{DP}+{C}_{IN}$
${\beta }_{a}$<2.32	${P}_{{f}_{a}}$>0.01	Severe	${C}_{R}+{C}_{E}+{C}_{DP}+{C}_{IN}+{C}_{L}+{C}_{IL}$

Here, the damage cost includes the cost of repair ${C}_{R}$, the loss of equipment ${C}_{E}$, the deferred production loss ${C}_{DP}$, the cost of injuries ${C}_{IN}$, the loss associated with fatality ${C}_{L}$, the indirect losses ${C}_{IL}$ related to the loss corresponding to platform collapse, an economic loss from functional disruption, and an environmental and social loss. Each of the following damage cost components is a function with respect to the damage index^15^. For deferred production loss, from Eq. (22).

22 $$ C_{DP} = 0.1 \times { }P_{P} \times { }T_{R} \times { }P_{R} $$

where ${P}_{P}$ is the current price of the platforms' product, ${T}_{R}$ is the estimated time to restore normal production, and ${P}_{R}$ is the platform's production rate. Here, it is assumed that the profit is 10% of ${P}_{P}$. The cost of injuries is determined using Eq. (23).

23 $$ C_{IN} = C_{1I} \times { }N_{I} $$

where ${C}_{1I}$ is the cost of an injury, and ${N}_{I}$ is the expected number of injured personnel. The loss associated with fatality is determined using Eq. (24).

24 $$ C_{L} = { }C_{1L} \times { }N_{D} $$

where ${C}_{1L}$ is the cost of a life lost, and ${N}_{D}$ is the expected fatalities. The annual secondary cost will be spent in the future but at different times, while the initial cost of the structure at present is not comparable to the future. Therefore, the annual secondary cost should be converted to the equivalent of the current rate using a certain discount rate. The interest rate for discounting is a rate that reflects an investor's opportunity cost of money over time; this means that an investor would want to achieve a return at least as high as that of his next best investment. Hence, the discount rate represents the investor's minimum acceptable rate of return. The cost of the secondary service of each model will be calculated by converting the cost of damage related to each year of life of the platform to their net value (*Pw*) and obtaining the total costs. Converting the cost of 25 years of service to the current value expressed as a coefficient in Eq. (25). If we multiply this equation by the total secondary cost in one year, the secondary cost in 25 years is obtained. For calculating the *Pw*, the discount rate (*d*) is determined. Thus, by calculating the total present value of the secondary cost and the initial cost, the LCC is determined. Finally, the optimum structure is achieved when the LCC is minimized.

25 $$ P_{w} = \frac{{\left( {1 + d} \right)^{LS} - 1}}{{d\left( {1 + d} \right)^{LS} }}/LS $$

## Case study

**Figure 2:**
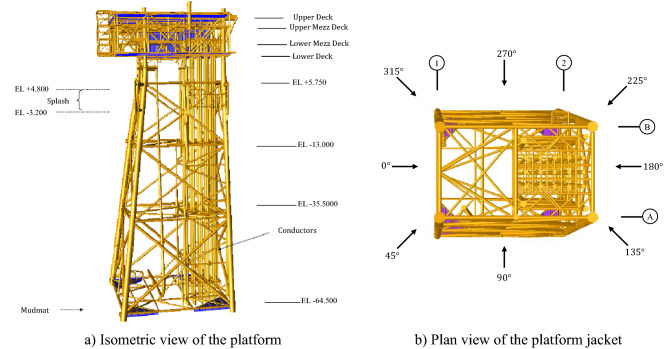
The offshore platform model.

The offshore platform in this study is a fixed metal base platform as shown in Fig. 2; it is located in the 19th phase of the South Pars gas region in the Persian Gulf. The structure consists of two parts; a jacket, and a deck. The jacket consists of 4 tilted bases with slopes of 1: 7 and 1: 8 and its weight is 2205.0 T. Here, only the pile members above the seabed are included, and the structure is assumed to be rigidly fixed at the seabed^50^. The weight of the deck is 1375.0 T, which is a five-story building and the height of each floor is 4.0 m. The dimensions of the decks are the same and all are 35.5 × 27.5; the distance between the bases at the base level or working level is 13.7 × 24 m^23^. The height codes of the jacket and deck floors in comparison to the lowest astronomical tide (LAT) are given in Table 3. Depending on the structural types, steel grades with different yield stresses are used, which are expressed based on the thickness of the element in Table 4. The diameter and thickness of the jacket elements vary depending on the type of base elements or horizontal and vertical bracing elements. The diameter and thickness of the elements are given in Tables 5 and 6. In the splash zone, i.e. between elevation − 3.20 m and + 4.80 m to LAT, a 6 mm corrosion allowance is considered for Jacket legs, vertical diagonal braces, etc. Nevertheless, no corrosion allowance is considered for the boat landing. The same philosophy extends to barge bumpers. The thickness corresponding to corrosion allowance is removed from the exterior surface of the tubular for calculation of stiffness and member and joint stresses. However, total thickness is used for weight calculation by overriding the densities or overriding cross-section area.

**Table 3:** Jacket and deck elevations.

Jacket		Deck	
Property	Elevations (m)	Property	Elevations (m)
Top of jacket	(+) 7.25	Upper deck and helideck	(+) 25.75
Jacket walkway level	(+) 5.75	Upper Mezz deck	(+) 21.85
Level-1	(−) 13	Lower Mezz deck	(+) 18.3
Level-2	(−) 35	Lower deck	(+) 14.25
Level-3	(−) 64.5	Drain deck	(+) 10
Mudline elevation	(−) 65	Height jacket	77
Pile stub elevation	(−)80	Height platform	90

**Table 4:** Specifications of the steel profiles for different structural components.

Steel grade	Yield stress (N/mm^2^)						Tensile strength (N/mm^2^)	
	Nominal thickness in mm							
	16 ≤	16 > ≤ 40	40 > ≤ 63	63 > ≤ 80	80 > ≤ 100	100 > ≤ 150	≤ 100	100 > ≤ 150
S235	235	225	215	215	215	195	340–470	340–470
S275	275	265	255	245	235	225	410–560	400–540
S355	355	345	335	325	315	295	490–630	470–630

**Table 5:** Structural specifications for the horizontal and legs members of the jacket.

Group of elements	Horizontal bracing							Legs		
Diameter (mm)	660			610				1755	1725	1665
Thickness (mm)	25.4	19.1	12.7	25.4	19.1	15.9	12.7	65	50	38

**Table 6:** Structural specifications for the vertical bracing members of the jacket.

Group of elements	Vertical bracing											
Diameter (mm)	864		762		660	610					508	
Thickness (mm)	38.1	25.4	19.1	12.7	38.1	38.1	25.4	19.1	15.9	12.7	38.1	25.4

Here, the wave loads on the offshore platform are calculated using SACS package^51^, which is an FE-based package, developed specifically for analyzing offshore platforms under conventional loads. Information about wave and wind characteristics is described in Table 7 and sea current characteristics are given in Table 8 for a 100-year return period. The 100-year maximum water depth for the in-place analysis is taken as LAT level from the seabed plus values of the mean highest high water and a 100-year storm surge as shown in Table 9. As environmental conditions such as tides vary according to the location of the structure, different geographical directions should be introduced. Here, the gravity load conditions, i.e. live and dead loads, and the harsh environmental load conditions such as wind, wave, and current including marine growth effects are considered the influential loadings in the design of the offshore platform.

**Table 7:** Summarizes wave and wind parameters used in the analysis.

Directions in the model	0°	45°	90°	135°	180°	225°	270°	315°
Wave from	SE	E	NE	N	NW	W	SW	S
Extreme storm wave height (m)	11.6	10.8	8.8	9.7	12.2	10.8	8.8	10.2
Extreme storm wave period (s)	10.8	10.4	9.6	10.0	11.0	10.4	9.5	10.2
Extreme wind speed 1-min (m/s)	35.2	36.0	34.9	35.6	36.7	35.6	33.0	33.4

**Table 8:** The following currents are considered for the design of the platform.

Current	Surface current	Mid-depth current	1.0 m above the seabed	0.5 m above the seabed
Current velocity (m/s)	1.28	1.28	0.78	0.71

**Table 9:** The 100-year maximum still water depth data for dynamic analysis.

Parameter	Value (m)							
	SE	E	NE	N	NW	W	SW	S
Water depth	66.6	66.5	66.4	66.4	66.6	66.5	66.4	66.5

According to API-RP2A, wind load is calculated in each direction by determining the shape coefficients and changes in wind speed at different altitudes. The calculation of the load due to the wave depends on the ratio of the length to the diameter of a part of the platform. For the considered jacket structure, the members will not modify the incident wave since the wavelength to the diameter ratio is higher than five. Wave forces acting on the jacket structure are calculated by Morison’s equation^52^. The surface roughness led by marine growth is referred to in calculating the wave force in Morison’s equation. In design, the marine growth effect according to Table 10 is considered in the platform elements. The vector sum of currents caused by tides and stormy conditions reaches the overall current. The total sea forces on the element are calculated by integrating the velocity profile. The dead load is from the combination of the self-weight of the deck, jacket structure, permanent equipment on the topside, etc. The weights of miscellaneous structural components, which are not part of the structural model, are input separately for each floor. The total weight of the non-generated items for all floors is 300.0 T. The weights of drilling and production equipment, the weight of chemical liquids, etc. are the components of the live loads. To simplify the modeling process, the deck is assumed to be ideally rigid in the floor and the loads on the deck are entered consistently at the highest level of the jacket. A summary of the loads is given in Tables 11 and 12. The design variables in the models are the thickness and outer diameter of the bases, and horizontal and vertical braces. The final load is calculated from the combination of 100% dead load, 50% live load, and the maximum severe environmental loads of the design with a 100-year return period in each geographical direction from 0° to 315°, separately.

**Table 10:** Increasing in the thickness of platform elements due to the effect of marine growth.

Thickness	From	To	Dry density
75 (mm) radial	EL (+) 2.0 m	EL (−) 6.0 m	1400 kg/m^3^
75 (mm) decreasing to 50 (mm) radially	EL (−) 6.0 m	Mudline

**Table 11:** Summary of the dead and live loads on deck.

Dead loads		Live loads	
Description	Weight (kN)	Description	Weight (kN)
Architectural, electrical, fire and safety, HVAC, instrumentation, mechanical-empty, mechanical- contents, piping-dry, piping-contents, X mass tree	15,800	Open area, laydown area, muster area, building area, drilling, production equipment, chemical liquids	5830

**Table 12:** Summary of the masses for the 100-years.

Item	Weight (kN)		
	X	Y	Z
Plate elements	2150	2150	2150
Plate element added mass	233	233	233
Member elements	55,112	55,112	55,112
Member element normal added mass	20,682	22,047	8506
Flooded member element entrapped fluid	5029	5029	5029
Load cases converted to weights	39,570	39,570	39,570
User-defined weights in dynamic analysis	1888	944	944

### Dynamic analysis

Performing a dynamic analysis, first, the equation directing the vibration of the platform is written using Eq. (26). In this equation, *F(t)* is the wave force in the direction of the wave motion, which is calculated from Morrison’s equation. *x* is the displacement of the structure in the direction of wave motion and as a function of time and water depth in the vicinity of the member. *C* is the damping of the structure, *K* is the stiffness of the whole structure and *M* is the total mass of the structure; the weight of the deck, and the weight of the plant organisms attached to the platform^53,54^.

26 $$ M\frac{{\partial^{2} x}}{{\partial^{2} t}} + C\frac{\partial x}{{\partial t}} + Kx = F\left( t \right) $$

**Figure 3:**
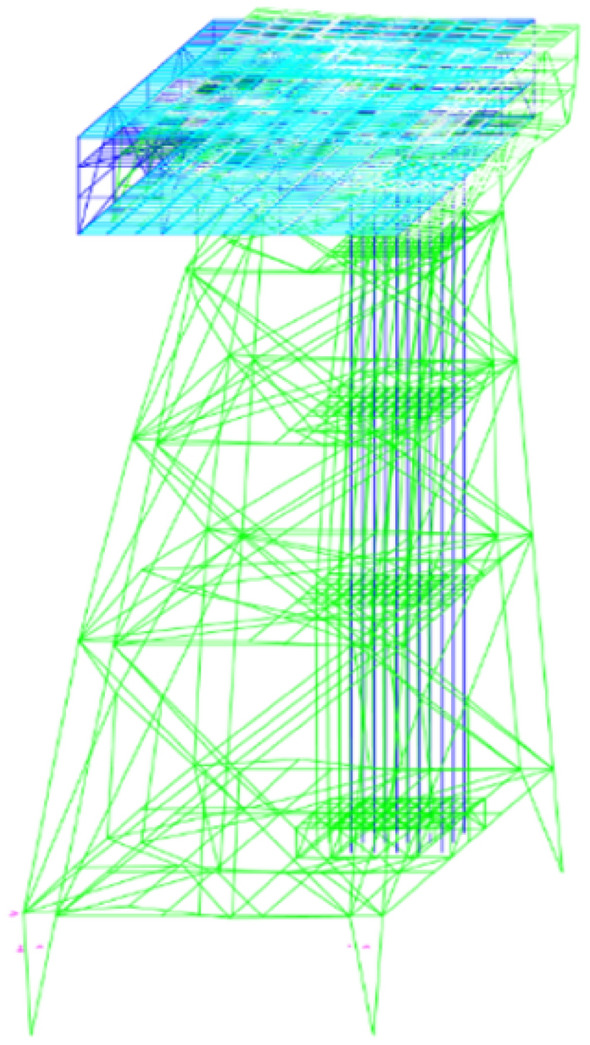
The first vibrating mode of the platform.

Due to the dynamic and stochastic nature of sea waves, the time history method is used to predict the response of waves where the dynamic behavior of the platform based on the application of a random wave with its various elements is considered a function of time^55^. Here, the time history is analyzed according to the DNV standard^56^ for a record of at least 1200 s with a time step of 0.25 s. According to the geographical conditions of the Persian Gulf and to simulate a random wave, Stoke's five-order theory and the John Swap spectrum are used. Figure 3 shows the platform under the first vibrating mode. For the first and second vibrating modes, the periods are respectively 9.71 s and 8.31 s, and the vibrational frequencies are respectively 0.101 (1/s) and 0.119 (1/s).

**Figure 4:**
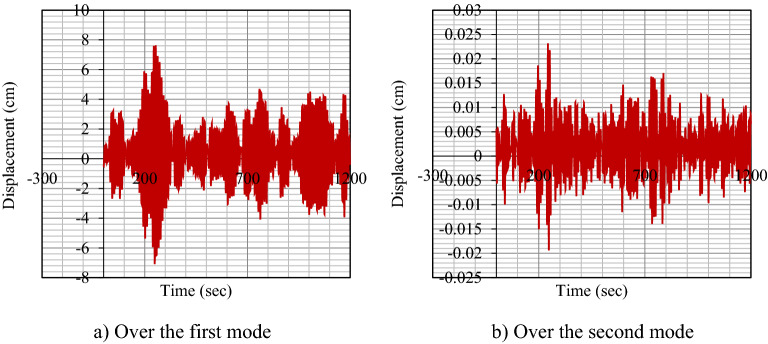
Dynamic response of the structures under the first and second modes.

Figure 4a,b shows the dynamic responses of the structure for the first two vibrational modes. As seen, the first mode is the dominant mode so it can be used for subsequent analyses.

### Determining the PoF and ${\mathbf{P}}_{{\mathbf{f}}_{\mathbf{a}}}$

**Figure 5:**
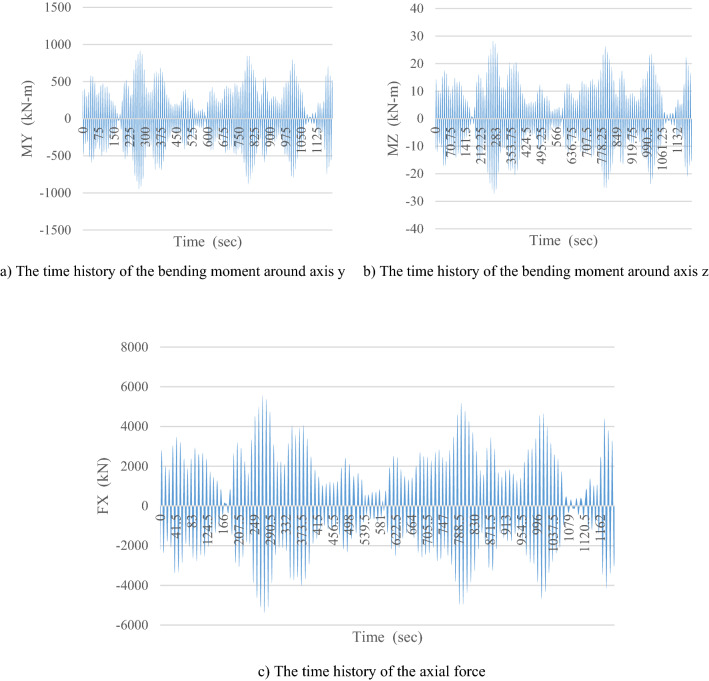
The time history of the axial force and bending moments over the y and z axes.

The reliability problem includes two components. The first component is the limit state function that defines the failure range^57^. This function consists of one or more random variables. The second component is the uncertainty variables, which are defined by a set of probabilistic distribution functions and determine the parameters associated with each distribution. To solve the reliability problem, the goal is to calculate the PoF based on the failure criterion. In addition, the platform element as the failure index is considered under the interaction of the axial force, the bending moment in two directions, elasticity modulus, and yield stress. This is worth mentioning that even the failure of a single component can cause the whole system to fail progressively. To determine the reliability index, it is necessary to specify first the performance function or the limit state function. As for the mutual behavior of waves around the element, the desired element may be stretched at one moment and compressed at another. Hence, for both tensile and compress states, it is necessary to involve two performance functions. This should also be noted that the study planned here does not consider a progressive collapse scenario where a single element loss may result in a series of successive failures. When the tubular elements are subjected to combined axial tension and bending, the boundary limit function for the elements can be calculated via Eq. (27)^58^.

27 $$ g = 1 - \left[ {\left( {\frac{{N_{Sd} }}{{N_{t, Rd} }}} \right) + \sqrt {\left( {\frac{{M_{y, Sd} }}{{M_{Rd} }}} \right)^{2} + \left( {\frac{{M_{z, Sd} }}{{M_{Rd} }}} \right)^{2} } } \right] $$

where *N*_*tRd*_, *M*_*Rd*_, and *N*_*Sd*_ are the axial force capacity in the tensile state, the bending capacity, and the amount of design axial tensile force the member, respectively. *M*_*y,Sd*_ and *M*_*z,Sd*_ are the design bending moments about member y-axis and bending moment about member z-axis, respectively. When tubular members are subjected to the combined axial compression and bending, the boundary limit function for the elements can be calculated via Eq. (28).

28 $$ g = 1 - \left( {\frac{{N_{Sd} }}{{N_{c,Rd} }} + \frac{1}{{M_{Rd} }}\sqrt {\left[ {\frac{{\dot{C}_{{m{\text{y}}}} M_{{{\text{y}},Sd}} }}{{\left[ {1 - \frac{{N_{Sd} }}{{N_{Ey} }}} \right]}}} \right]^{2} + \left[ {\frac{{\dot{C}_{{m{\text{z}}}} M_{{{\text{z}},Sd}} }}{{\left[ {1 - \frac{{N_{Sd} }}{{N_{Ez} }}} \right]}}} \right]^{2} } } \right) $$

where *N*_*cRd*_ is the capacity of the axial force in the compression state. When the platform element is under a compressive axial load, the deformation and the buckling effects on the performance function are considered. Also, $\dot{Cm}$ is the co-existence coefficient of the maximum moment with secondary moments and *N*_*E*_ is the Euler loading moments. The two introduced performance functions are in the state of safe when the value *g* is greater than zero and in the state of failure when this value is less than zero. The boundary state between failure and safety occurs when this function is zero. Then the statistical parameters of the random variables are specified. Here, three parameters of diameter, thickness, and length of platform members are considered definitively due to the careful supervision of consulting companies on the construction of the members; but the axial force, bending moment around y and z axes, modulus of elasticity, and yield stress are considered as random variables^59^. To determine the statistical parameters related to the axial force and flexural anchors of the members, the structure is dynamically analyzed in the presence of wave forces over a random manner. For this purpose, one 0.3-h storm simulation for the water surface elevation is produced to capture the statistical properties of extreme sea conditions. After simulation of the irregular sea and making surface elevations, time histories of the applied hydrodynamic loads for elements in each geographical direction from 0° to 315°, separately are produced. Time histories of the structure response are achieved by using the numerical time-domain integration of the equation of motion by application of the time histories of the applying load. To this end, the random wave module of the SACS program is used and structural dynamic responses are calculated at each 0.25-s interval, which is shown in the graphs; the demanded outputs, including the time history of ${M}_{y}$, ${M}_{z}$ and ${F}_{X}$ where they are introduced as random variables for each structural element. For example, the time history of ${M}_{y}$, ${M}_{z}$ and ${F}_{X}$ for element number of 6 in geographical direction 180° are shown in Fig. 5a–c.

After introducing the random variables, the type of probability distribution function should be specified. To select the best distribution for the random variables, the data related to these variables are analyzed using Easy-Fit software. For this purpose, three different distribution functions the Normal, Log-Normal, and Weibull were selected for each random variable. After extracting the data distribution function and histogram, a Chi-square test comparison was used and the best probability distribution was selected and scored for each random variable. It found that for axial force and flexural anchor toward the z-axis, the Weibull distribution function and the flexural anchor around the y-axis are the Log-Normal distribution functions. Also, according to studies conducted concerning a structure’s reliability analysis, for steel yield stress, the Log-Normal distribution function is determined by a 10% variation coefficient with a 355 MPa average. For the elastic modulus of steel, a Normal distribution function is considered with a 25% variation coefficient with a 2 × 10^5^ MPa average^60^.

Finally, using MATLAB and based on the FORM methods, programmed reliability, and the, β are calculated followed by the PoF of each element; here, the PoF for each element under tensile and compressive performance (see Eqs. 27, 28) is determined according to Eqs. (5–11), where for each element minimum value is considered as the critical reliability index. Subsequently, ${\beta }_{a} \mathrm{and}$ ${P}_{{f}_{a}},$ for each element, and a period of design lifetime is calculated from Eqs. (12–15)^17^. Here, *β* was used for evaluating the LCC and thus the PoF.

### LCC calculation

For the LCC calculation, assumptions regarding various stages via development, transportation, installation, maintenance, assembly, and decommissioning cost for multiple designs are made as discussed hereafter. CAPEX is the initial investment and is calculated for each structural design using Eq. (17) where *a*, is 1.064. In this study, it is assumed that the manufacturing cost is 94% of the CAPEX^45^.

Steel prices do vary randomly between countries and geographic locations and depend on other external factors. The base marine quality treated S335 steel price is set to $1200/ton. In this study, the manufacturing cost is based on an addition of 400% to the material cost^61^. The total cost of constructing the platform consists of the cost of the base part of the platform (jacket) plus the cost of the upper part of the platform (deck), where the cost of the upper part includes the cost of equipment, installation, transportation, and commissioning of equipment and the construction and installation of the deck skeleton; and the base cost includes the cost of construction and installation of the jacket structure plus the cost of the sea section. Also, the cost of the offshore sector (shipping and installation), on average, is 40–45% of the total cost of construction and installation of jackets. Due to the differences in the price of jacket or deck constructions, quality of materials, quality of work, and execution time in different companies' offshore platforms, several companies in the industry of manufacturing fixed offshore platforms in waters with an average depth of 65 m were questioned; the average time required for construction, transportation, installation, and commissioning for the base part of the platform was 1095 days and the offered prices were close to each other; the results are given in Tables 13, 14 and 15. To estimate the price of wellhead equipment in terms of production capacity, according to Table 15, they are divided into two categories. In Table 15, a million standard cubic feet per day (MMSCFD) is a unit of measurement for natural gas. One MMSCFD equals 1180 m^3^/h.

**Table 13:** Average cost of construction, transportation, and installation of the deck platform.

Deck weight (ton)	$$w<1000$$	$$1000\le w<1500$$	$$1500\le w<2000$$	$$2000\le w<2500$$	$$2500\le w$$
Unit cost ($/kg)	25	20	18	16	12

**Table 14:** Average cost of construction, transportation, and installation of the jacket platform.

Jacket weight (ton)	w < 1000	1000 ≤ w < 2000	2000 ≤ w < 3000	3000 ≤ w < 4000	4000 ≤ w
Unit manufacturing cost ($/kg)	11	9	8	7	5
Transportation and installation cost ($/kg)	5	4	3	2	1.75

**Table 15:** Average cost of equipment in production platforms.

The main equipment of the production platform	Production capacity (MMSCFD)	Cost of equipment (M$)
Wellhead system, HP/LP flare system, gas/water separation, electric power generation system, F&G, all substations, all control systems, HVAC, all utilities, bulk material, and other contractor items	500	87
	1000 and 880	106

Table 13 shows that the average weight of the deck is 1375 T, and the cost of construction, transportation, and installation is 20$/kg. It can be concluded from Table 15 that, considering that the platform is of the good production type with MMSCFD intermediate power (880–1000), the average cost of equipment is estimated to be 106 M$. Here, five models are designed and named from S1–S5. To estimate the CAPEX calculation for each alternative, they are divided into 6 categories as given in Table 16.

**Table 16:** The assumed auxiliary values for the CAPEX for each alternative S_1_ to S_5_.

Structural model	${C}_{ed}/{C}_{m}$	${C}_{ga}/{C}_{m}$	${C}_{gs}/{C}_{m}$	${C}_{yf}/{C}_{m}$	${C}_{cle}/{C}_{m}$	${C}_{pt}/{C}_{m}$
$S1$	0.04722	0.02411	0.01473	0.1651	0.71481	0.03351
S2	0.04912	0.02509	0.01532	0.17202	0.71643	0.03491
S3	0.05106	0.02608	0.01593	0.17881	0.71805	0.03628
S4	0.05313	0.02714	0.01657	0.18606	0.71967	0.03775
S5	0.05533	0.02826	0.01726	0.19378	0.72129	0.03932

Operational expenses arise from performing normal business operations. The operational cost consists of annual inspection, repair costs, etc. The lifespan cost of repair and maintenance services during a platform operation is estimated by Eq. (19) where it is assumed that *b* is 10% of the CAPEX per annum^62^. Table 17, for example, shows some parameters for operating and maintaining cost evaluation.

**Table 17:** Some parameters for operating and maintaining cost evaluation.

Parameter	${C}_{re}$	${C}_{cp}$	${C}_{rti}$	${C}_{rpw}$	${C}_{hl}$	${C}_{flo}$
%of the initial cost	0.0698	0.0483	0.0994	0.0391	0.0271	0.695

The RISKEX is the failure risk expenditure due to an extreme environmental load. Here the secondary risk cost of the lifetime is estimated via Eq. (20). According to the division of annual secondary cost based on the reliability index, the annual loss is calculated by Eq. (21). As there is no released report regarding the effect of oil or gas leaking on the ecosystem of the Persian Gulf, here, to facilitate the calculation process, the data released after the Mexico Gulf event is employed; see Table 18. Parameters used in Eq. (22), P_R_ = 300 million *ft*^*3*^ of sour gas and 30 million *ft*^*3*^ of combustible gas per day, T_R_ = 27 months, P_P_ = 60,000 USD per 30 million *ft*^*3*^ of sour gas, and 508,889 USD per *ft*^*3*^ of combustible gas^48^. Here, are the parameters in Eqs. (23 and 24), *N*_*D*_ and *N*_*1*_, are related to the number of injured and casualties, respectively, the people present on the platform are supposed to be 10 people^15^. The discount rate (d) in this study according to its location in Iran is assumed to be 0.10.

**Table 18:** Some parameters for damage cost evaluation.

Parameter	${C}_{R}$	${C}_{E}$	${C}_{DP}$	${C}_{1I}$	${C}_{IN}$	${C}_{1L}$	${C}_{L}$	${C}_{IL}$
% of the initial cost	17.2	68.0	1800.0	0.008	0.08	0.075	0.75	9060.0

For each structural plan, the LCC is calculated according to Eqs. (16–25). Therefore, in structural design, a plan is acceptable if it creates a balance between the initial costs with the cost of possible damage.

## Models and results

**Figure 6:**
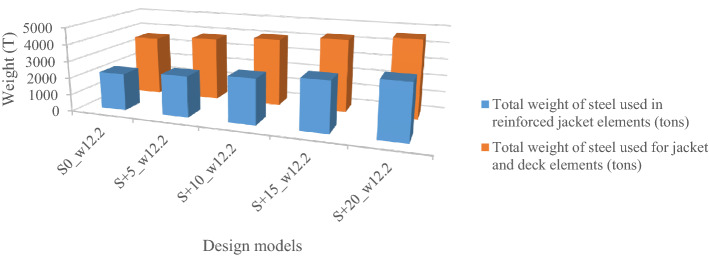
The amount of steel consumed in each model.

Five models are now designed; a model based on the codes’ requirements and the rest four models for more than the requirements up to a 20% increase. The models are abbreviated as S_x__W_y_ in which x is the percentage increase and y is the maximum dominant wave height associated with the 100-year return period. For example, the S + 10_w12.2 model is the maximum dominant wave height for the northwest with a height of 12.2 m and 10% reinforcement of all jacket elements more than the requirements. Hereafter, we define the models S0_w12.2, S + 5_W12.2, S + 10_W12.2, S + 15_W12.2, and S + 20_W12.2, respectively as S1–S5. The results are given in Fig. 6.

### Investigating the PoF and LCC of the models

**Figure 7:**
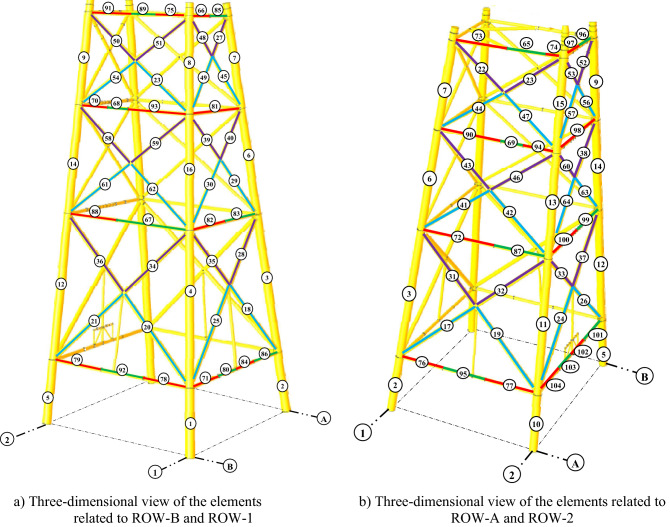
Three-dimensional view of the jacket elements.

**Figure 8:**
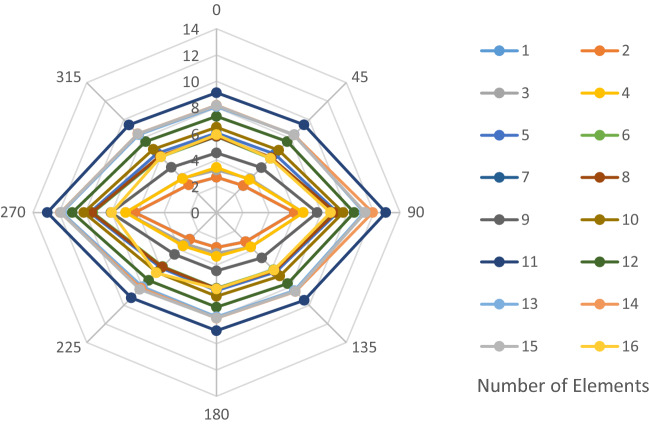
Reliability index of leg elements in different directions.

**Figure 9:**
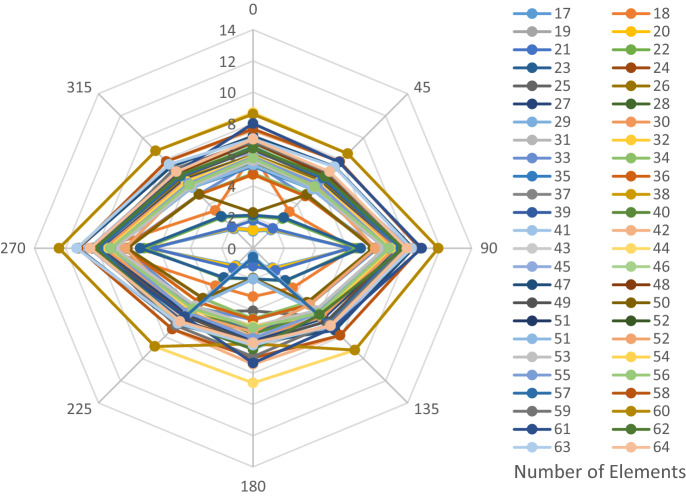
Reliability index of vertical bracing elements in different directions.

**Figure 10:**
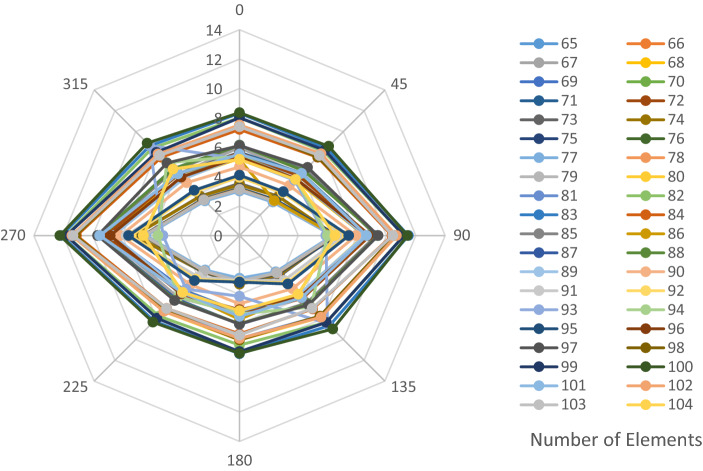
Reliability index of horizontal bracing elements in different directions.

**Figure 11:**
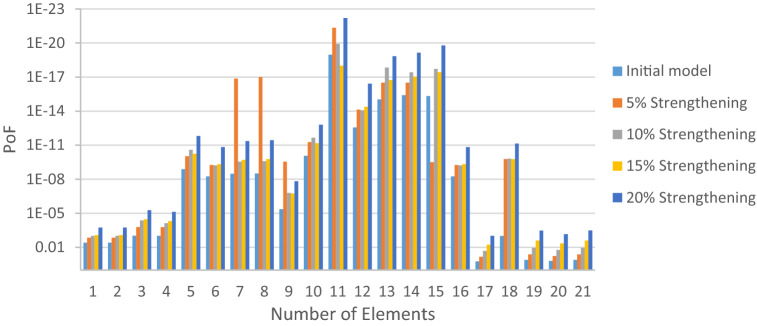
Effect of reinforcement on reducing PoF of Elements 1 to 21.

**Figure 12:**
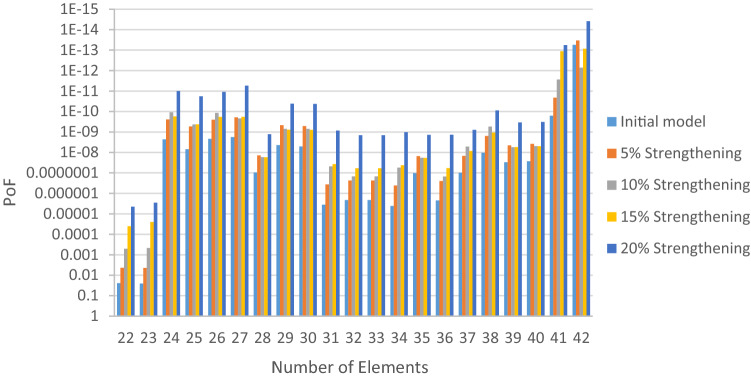
Effect of reinforcement on reducing the PoF of Elements 22 to 42.

**Figure 13:**
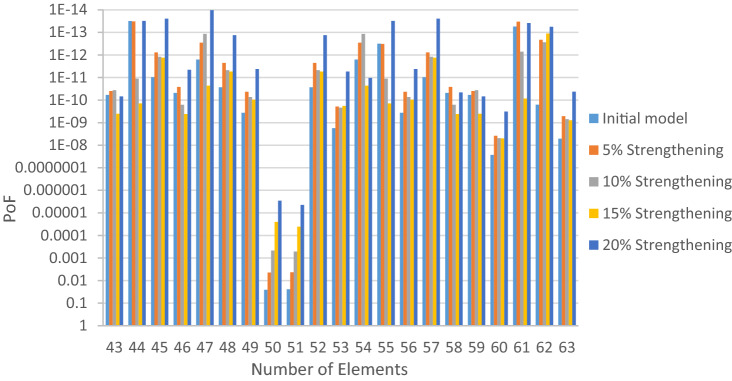
Effect of reinforcement on reducing the PoF of Elements 43 to 63.

**Figure 14:**
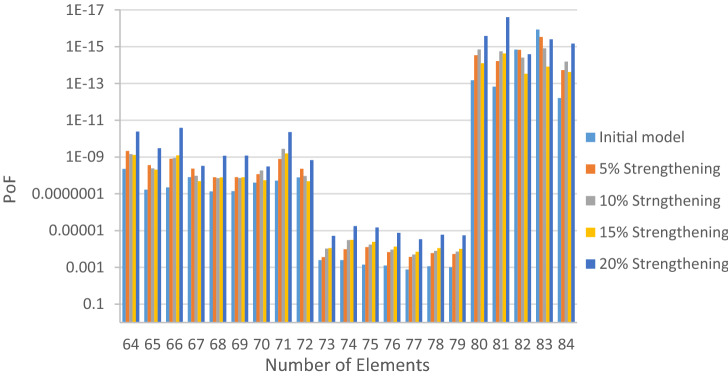
Effect of reinforcement on reducing the PoF of Elements 64 to 84.

**Figure 15:**
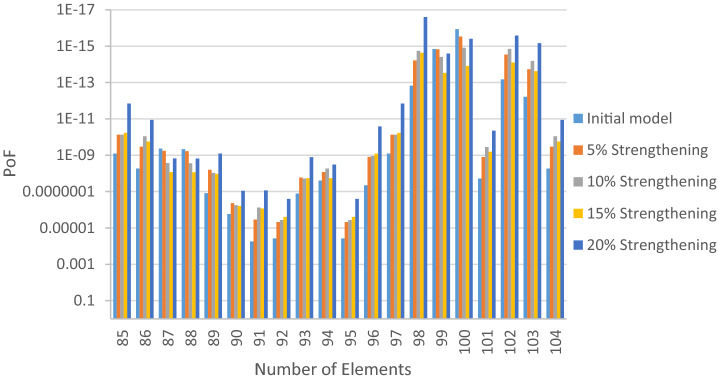
Effect of reinforcement on reducing the PoF of Elements 85 to 104.

The structural elements are shown in Fig. 7. In the code-based model the reliability index, vertical, horizontal, and base bracing elements in all directions are presented in Figs. 8, 9 and 10; it is found that the reliability index in the northwest direction has the lowest value. Hence, it is the critical direction; the reason is that the wave height in the direction of 180 degrees is higher than in other directions. Now, the possibility of failure under the applied loads and over the critical direction is determined using FORM for the structural elements. The corrosion percentage, as pointed out earlier, is also involved for the jacket legs, and vertical and diagonal braces. As well, the marine growth effect, which can increase the applied load on the structure, is considered while the dynamic analysis being performed. Therefore, the case studied for each stage of retrofitting over the critical direction is re-analyzed to account for new axial forces and bending moments. Accordingly, new statistical parameters and the type of probability distribution function are all re-evaluated. The reliability index and the PoF for each element are then accounted for. The reinforcement effect on reducing the PoF over the critical direction is shown in Figs. 11, 12, 13, 14 and 15; the increase in the reinforcement of the elements in each model is due to the effect of simultaneous changes in the thickness and outer diameter of the pipe.

**Figure 16:**
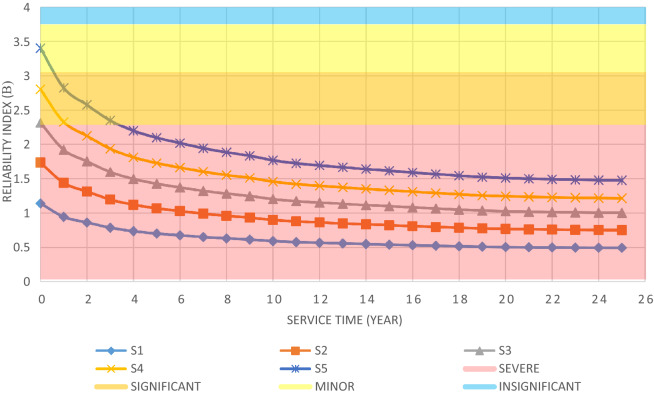
Variation of reliability index of design models with service time for Element 19.

**Figure 17:**
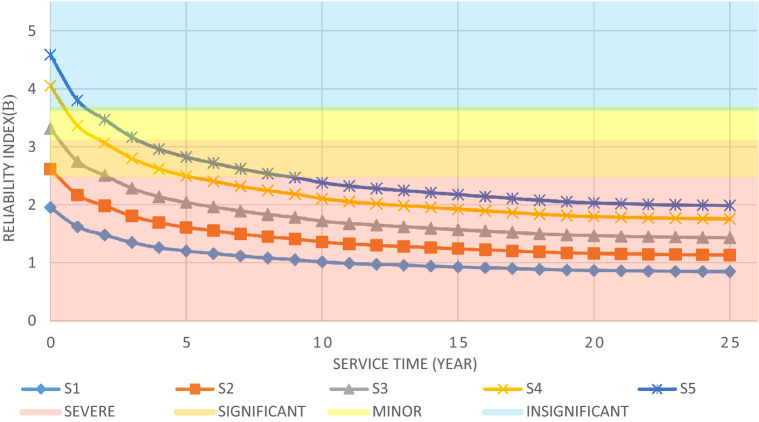
Variation of reliability index of design models with service time for Element 23.

**Figure 18:**
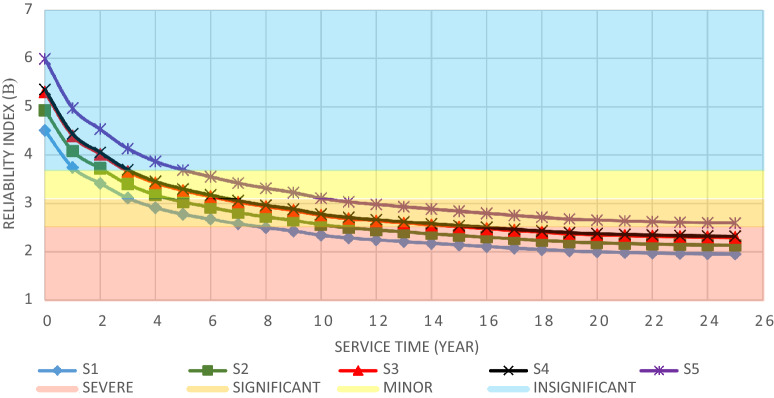
Variation of reliability index of design models with service time for Element 34.

**Figure 19:**
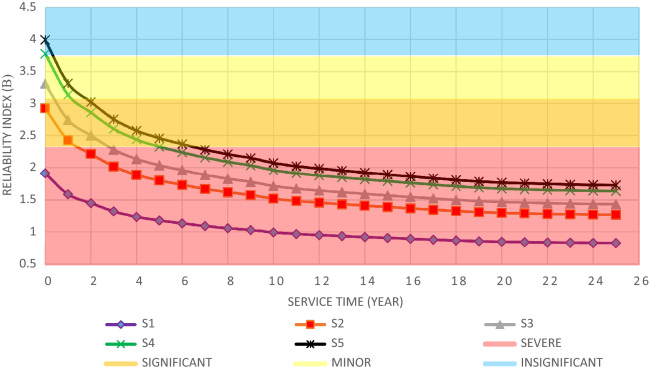
Variation of reliability index of design models with service time for Element 50.

From Figs. 11, 12, 13, 14 and 15, it is observed that in the code-based model, the possibility of damage to the horizontal and base bracing elements is in the range of minor to medium. It is observed that in the code-based model, the PoF in vertical bracing elements in the tidal zone and close to the seabed is in the range of severe to moderate consequences. Gradually, with the strengthening up to 20%, an increase in the confidence index and a decrease in the PoF is seen. The above figures show that some elements have higher PoF than others. It can be argued that the elements close to the joints increase due to the effects of punch cutting, axial force, and bending moment, and as a result, we expect a higher PoF. It should also be noted that reinforcement takes place in elements that exceed the target reliability index; otherwise, there is no need to reinforce the elements. Hence, to determine the annual damage of S1–S5, the PoF and the reliability index for each element should be determined. To do that, a computer program is written to determine ${P}_{{f}_{a}}$ and ${\beta }_{a}$ for the duration of service time; the results are shown in Figs.16, 17, 18 and 19. As illustrated, the reliability index of elements decreases while the service time increases. Inspection, repair, and maintenance might be carried out during the service time of the offshore platform. When the annual reliability index for each element approaches the minimum annual reliability index (i.e. 2.32), the repair and maintenance actions can now be scheduled. The complete information is given in “Appendix A”.

From Figs. 16, 17, 18 and 19, it is understood that the scheduled time for a repair differs from one element to another. It is also understood that for the code-based model, the inspection time is sooner than that of other models; yet, as time passes and the elements are being retrofitted up to 20%, the reliability index increases, and the need for retrofitting decreases. For example, for element number 23, there is a need to repair for the years 1st, 3rd, 7th, and 11th.

**Figure 20:**
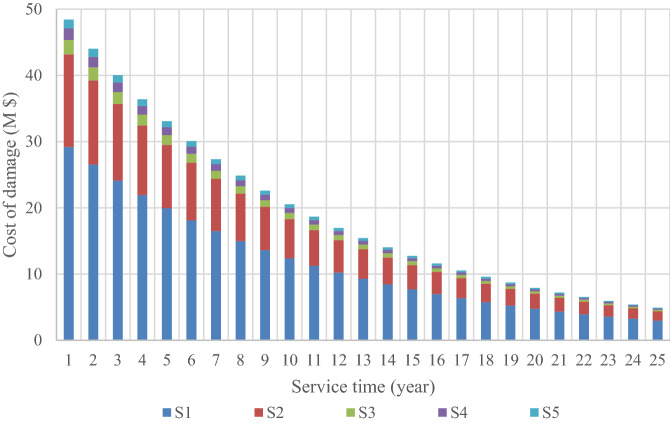
Comparison of the current cost of design model damage over 25 years.

**Figure 21:**
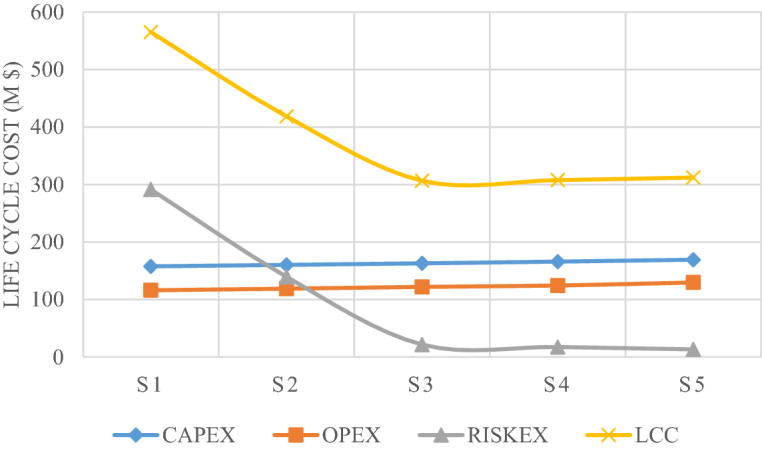
LCC analysis of the models.

Calculating the annual damage for S1–S5 allows us to get the secondary cost of that model for one year of the platform’s life. We can see that the secondary cost reduces by increasing the reinforcement of each model compared to the original model. The number of models on the horizontal axis is equivalent to a new design, which shows the reinforcement of the jacket elements. This damage value for 25 years’ service for each model is shown cumulatively in Fig. 20. The LCC of each structural model is obtained by calculating the secondary and primary costs from each model and converting the total life cost to this value; see Fig. 21.

According to Fig. 20, in the first years, the cost of damage to the structure in all models is much higher than in the last years of service; this can be correlated to the cost of annual damage with a discount rate that becomes equivalent to that at present. As shown in Fig. 21, a structure designed for lower loads will cost less to build, while the expected failure cost will be higher; with a slight increase in the initial cost, the total cost decreases significantly over the lifetime to where the other two curves intersect. It can be concluded that the design based on the minimum LCC is optimized for the S3 model.

### Comparing the results

**Figure 22:**
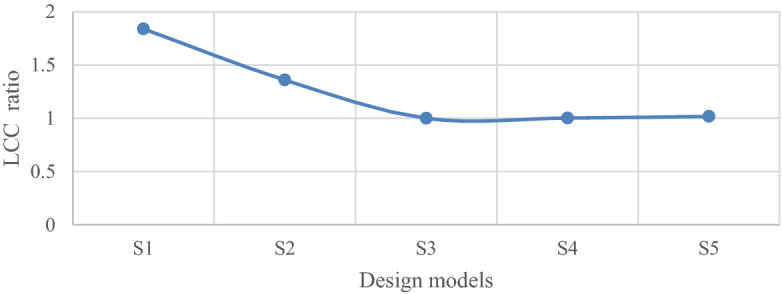
LCC ratio of each model to the optimal model.

**Figure 23:**
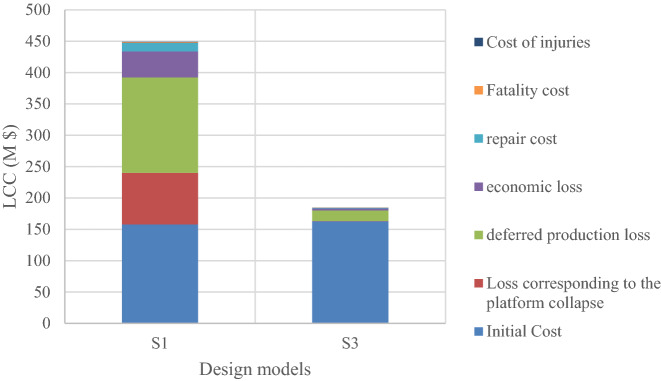
Primary and secondary costs over the lifetime service for S1 and S3 models.

It is observed that the S3 model with a minimized LCC is obtained by a 10% increase in the initial loads. This model has a 45.7% less LCC than the code-based model. Figure 22 shows the LCC ratio of each model to the optimal model (i.e. S3). Figure 23 compares the initial and secondary costs of the S1 and S3 models separately.

**Figure 24:**
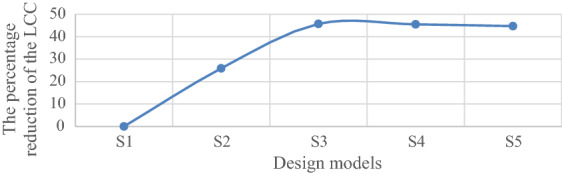
Comparing the percentage reduction in the LCC of the optimal model with other models.

The rate of cost reduction and increase of consumed steel of each model are now compared. As shown in Fig. 24, the percentage reduction in the LCC of the S5 model is about 44.7%; however, with the increase of reinforcement of the elements more than the defined amount up to 10%, the optimal model has a lower percentage reduction, i.e. 45.7%, compared to the original model.

**Figure 25:**
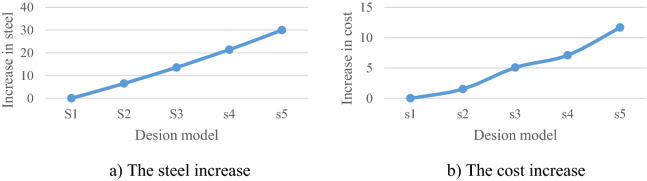
Comparing the models in terms of their cost increase and steel consumption.

Also, the percentage increase in steel consumption of sections in the S3 compared to the S1 is 13.56%, and the percentage increase in steel consumption of S2, S4, and S5 is 6.49%, 21.37%, and 30.0%, respectively. The reason for the additional steel in elements is due to the effect of increasing the thickness and diameter at the same time. Figure 25a shows the percentage increase in steel consumption and Fig. 25b shows the percentage increase in the cost of steel consumption; this shows the percentage increase in the cost of steel in S3 is 5% and the rate of increase in the cost of steel in S2, S4, and S5 are 1.51%, 7.1%, and 11.68%, respectively.

### Optimal design parameters

This section presents the solution of the optimal design parameters that yield minimum LCCs under the defined case study. Modified sections for the fixed offshore platform structure for S1 and S3 based on the changes in the reliability index are given in Table 19.

**Table 19:** Modified sections for the S1 and S3 models.

	Number of elements	Initial model		Optimal model	
		Diameter (mm)	Thickness (mm)	Diameter (mm)	Thickness (mm)
Legs	1-2-3-4	1755	65	1930.5	71.5
	5-6-7-8-16	1725	50	1897.5	55
	9-10-11-12-13-14-15	1665	38	1831.5	41.8
Vertical bracing	19-21	864	38.1	950.4	41.9
	17-20-31-32-34-36		25.4		27.94
	24-25-26	762	19.1	838.2	21
	18-28-33-35-37		12.7		13.97
	22-47	660	38.1	726	41.9
	48-49-50-51-55	610	38.1	671	41.9
	23-27		25.4		27.94
	29-30-39-40-54		19.1		21
	41-42-43-44-45-46-58-59-61-62		15.9		17.49
	38-60-63-64		12.7		13.97
	52-53	508	38.1	558.8	41.9
	56-57		25.4		27.94
Horizontal bracing	102	660	25.4	726	27.94
	71-77-78-92-79-93-94-103		19.1		21
	67-72-76-80-82-84-86-87-88-101-104		12.7		13.97
	66-68-74-81-91-96-97-98	610	25.4	671	27.94
	69-90		19.1		21
	65-73-75-89		15.9		17.49
	70-83-85-95-99-100		12.7		13.97

## Conclusion

Offshore platforms are important infrastructure in which any disruption over the lifetime use can impose considerable costs on their stakeholders. It is hence of vital importance to monitor their lifetime behaviors from different perspectives. While many studies have, to date, addressed the lifetime costs of offshore platforms or their loadings reliability, there are rare studies that have employed a probabilistic-based approach to the life-cycle cost (LCC) analysis of these structures. This concern was addressed in the current study.

A fixed offshore platform was designed in compliance with the requirements of API-RP2A-WSD, DNV, and NORSOK standards. Field surveys related to the platform in the Persian Gulf at a depth of 65 m were done. The environmental loads with a 100-year return period were used and a time history dynamic analysis was performed. The failure index was considered as jacket elements under axial force interaction and flexural anchor in two directions. Overall, five models were designed based on the same wave loads and over a critical direction; one model was designed based on the code’s requirements, and the rest for up to 20% increase to the requirements, were designed. The optimal model based on the minimum LCC was determined by changing the amplification of the elements of each model in the critical direction by the first-order reliability model (FORM). The criterion for determining the damage to the elements of each model was its placement in each level of failure outcome according to the range of the reliability index. Finally, a method for determining the optimal design parameters based on the minimum LCC was proposed. The following results were achieved.

- The model designed as per the codes’ regulations was not cost-effective over the lifetime; to achieve the optimal design, it was necessary to use a coefficient to change the reinforcement of the jacket elements. The results showed that this coefficient is bigger than one.
- The optimal model was obtained by increasing the reinforcement in terms of the external diameter and thickness of the elements up to 10% more than the code-based amounts.
- The rate of LCC reduction in the optimum model compared to the code-based model was 45.7%; while the rate of increase in the initial cost for the optimum model was 5% and the rate of increase in the steel consumption was about 13.56%.
- With an increase in the reinforcement of the elements more than the required one, the LCC gradually decreased. The LCC ratio in the code-based model to the optimum model was calculated as 1.84%

The probabilistic-based approach for the current study shows that the first inspection for each element differs from one to another. It can be said that the need for an inspection for elements that have a closer reliable index to the annual reliability index starts from the first year; as time passes and the elements are being retrofitted, this need decreases. This should also be noted that the work presented here was not to address a scenario under which the degradation of strength in the connections and the fatigue due to the impact loads are the cases. The proposed procedure in this study for determining the optimal design parameters and reliability would be meaningful and applicable to the development of an offshore platform from the conceptual design stage.

## Supplementary Information

Supplementary Information.

## Data Availability

Data are available from the authors upon reasonable request and with permission from http://library.aut.ac.ir. Please contact lib_office@aut.ac.ir.
