# Supplementary material for: A probabilistic approach to lifetime design of offshore platforms
Source: Sci Rep. 2023 May 2;13:7101. doi: 10.1038/s41598-023-34362-x (PMC10154351; doi:10.1038/s41598-023-34362-x)
Supplement: Supplementary file 1 — Supplementary Information. [file 41598_2023_34362_MOESM1_ESM.docx]

**Appendix A:** **Variation of reliability index of design models**

|  |
| --- |
| **Fig. A1: Variation of reliability index of design models with service time for element 1** |
|  |
| **Fig. A2: Variation of reliability index of design models with service time for element 2** |
|  |
| **Fig. A3: Variation of reliability index of design models with service time for element 3** |
|  |
| **Fig. A4: Variation of reliability index of design models with service time for element 4** |
|  |
| **Fig. A5: Variation of reliability index of design models with service time for element 5** |
|  |
| **Fig. A6: Variation of reliability index of design models with service time for element 6** |
|  |
| **Fig. A7: Variation of reliability index of design models with service time for element 7** |
|  |
| **Fig. A8: Variation of reliability index of design models with service time for element 8** |
|  |
| **Fig. A9: Variation of reliability index of design models with service time for element 9** |
|  |
| **Fig. A10: Variation of reliability index of design models with service time for element 10** |
|  |
| **Fig. A11: Variation of reliability index of design models with service time for element 11** |
|  |
| **Fig. A12: Variation of reliability index of design models with service time for element 12** |
|  |
| **Fig. A13: Variation of reliability index of design models with service time for element 13** |
|  |
| **Fig. A14: Variation of reliability index of design models with service time for element 14** |
|  |
| **Fig. A15: Variation of reliability index of design models with service time for element 15** |
|  |
| **Fig. A16: Variation of reliability index of design models with service time for element 16** |
|  |
| **Fig. A17: Variation of reliability index of design models with service time for element 17** |
|  |
| **Fig. A18: Variation of reliability index of design models with service time for element 18** |
|  |
| **Fig. A19: Variation of reliability index of design models with service time for element 19** |
|  |
| **Fig. A20: Variation of reliability index of design models with service time for element 20** |
|  |
| **Fig. A21: Variation of reliability index of design models with service time for element 21** |
|  |
| **Fig. A22: Variation of reliability index of design models with service time for element 22** |
|  |
| **Fig. A23: Variation of reliability index of design models with service time for element 23** |
|  |
| **Fig. A24: Variation of reliability index of design models with service time for element 24** |
|  |
| **Fig. A25: Variation of reliability index of design models with service time for element 25** |
|  |
| **Fig. A26: Variation of reliability index of design models with service time for element 26** |
|  |
| **Fig. A27: Variation of reliability index of design models with service time for element 27** |
|  |
| **Fig. A28: Variation of reliability index of design models with service time for element 28** |
|  |
| **Fig. A29: Variation of reliability index of design models with service time for element 29** |
|  |
| **Fig. A30: Variation of reliability index of design models with service time for element 30** |
|  |
| **Fig. A31: Variation of reliability index of design models with service time for element 31** |
|  |
| **Fig. A32: Variation of reliability index of design models with service time for element 32** |
|  |
| **Fig. A33: Variation of reliability index of design models with service time for element 33** |
|  |
| **Fig. A34: Variation of reliability index of design models with service time for element 34** |
|  |
| **Fig. A35: Variation of reliability index of design models with service time for element 35** |
|  |
| **Fig. A36: Variation of reliability index of design models with service time for element 36** |
|  |
| **Fig. A37: Variation of reliability index of design models with service time for element 37** |
|  |
| **Fig. A38: Variation of reliability index of design models with service time for element 38** |
|  |
| **Fig. A39: Variation of reliability index of design models with service time for element 39** |
|  |
| **Fig. A40: Variation of reliability index of design models with service time for element 40** |
|  |
| **Fig. A41: Variation of reliability index of design models with service time for element 41** |
|  |
| **Fig. A42: Variation of reliability index of design models with service time for element 42** |
|  |
| **Fig. A43: Variation of reliability index of design models with service time for element 43** |
|  |
| **Fig. A44: Variation of reliability index of design models with service time for element 44** |
|  |
| **Fig. A45: Variation of reliability index of design models with service time for element 45** |
|  |
| **Fig. A46: Variation of reliability index of design models with service time for element 46** |
|  |
| **Fig. A47: Variation of reliability index of design models with service time for element 47** |
|  |
| **Fig. A48: Variation of reliability index of design models with service time for element 48** |
|  |
| **Fig. A49: Variation of reliability index of design models with service time for element 49** |
|  |
| **Fig. A50: Variation of reliability index of design models with service time for element 50** |
|  |
| **Fig. A51: Variation of reliability index of design models with service time for element 51** |
|  |
| **Fig. A52: Variation of reliability index of design models with service time for element 52** |
|  |
| **Fig. A53: Variation of reliability index of design models with service time for element 53** |
|  |
| **Fig. A54: Variation of reliability index of design models with service time for element 54** |
|  |
| **Fig. A55: Variation of reliability index of design models with service time for element 55** |
|  |
| **Fig. A56: Variation of reliability index of design models with service time for element 56** |
|  |
| **Fig. A57: Variation of reliability index of design models with service time for element 57** |
|  |
| **Fig. A58: Variation of reliability index of design models with service time for element 58** |
|  |
| **Fig. A59: Variation of reliability index of design models with service time for element 59** |
|  |
| **Fig. A60: Variation of reliability index of design models with service time for element 60** |
|  |
| **Fig. A61: Variation of reliability index of design models with service time for element 61** |
|  |
| **Fig. A62: Variation of reliability index of design models with service time for element 62** |
|  |
| **Fig. A63: Variation of reliability index of design models with service time for element 63** |
|  |
| **Fig. A64: Variation of reliability index of design models with service time for element 64** |
|  |
| **Fig. A65: Variation of reliability index of design models with service time for element 65** |
|  |
| **Fig. A66: Variation of reliability index of design models with service time for element 66** |
|  |
| **Fig. A67: Variation of reliability index of design models with service time for element 67** |
|  |
| **Fig. A68: Variation of reliability index of design models with service time for element 68** |
|  |
| **Fig. A69: Variation of reliability index of design models with service time for element 69** |
|  |
| **Fig. A70: Variation of reliability index of design models with service time for element 70** |
|  |
| **Fig. A71: Variation of reliability index of design models with service time for element 71** |
|  |
| **Fig. A72: Variation of reliability index of design models with service time for element 72** |
|  |
| **Fig. A73: Variation of reliability index of design models with service time for element 73** |
|  |
| **Fig. A74: Variation of reliability index of design models with service time for element 74** |
|  |
| **Fig. A75: Variation of reliability index of design models with service time for element 75** |
|  |
| **Fig. A76: Variation of reliability index of design models with service time for element 76** |
|  |
| **Fig. A77: Variation of reliability index of design models with service time for element 77** |
|  |
| **Fig. A78: Variation of reliability index of design models with service time for element 78** |
|  |
| **Fig. A79: Variation of reliability index of design models with service time for element 79** |
|  |
| **Fig. A80: Variation of reliability index of design models with service time for element 80** |
|  |
| **Fig. A81: Variation of reliability index of design models with service time for element 81** |
|  |
| **Fig. A82: Variation of reliability index of design models with service time for element 82** |
|  |
| **Fig. A83: Variation of reliability index of design models with service time for element 83** |
|  |
| **Fig. A84: Variation of reliability index of design models with service time for element 84** |
|  |
| **Fig. A85: Variation of reliability index of design models with service time for element 85** |
|  |
| **Fig. A86: Variation of reliability index of design models with service time for element 86** |
|  |
| **Fig. A87: Variation of reliability index of design models with service time for element 87** |
|  |
| **Fig. A88: Variation of reliability index of design models with service time for element 88** |
|  |
| **Fig. A89: Variation of reliability index of design models with service time for element 89** |
|  |
| **Fig. A90: Variation of reliability index of design models with service time for element 90** |
|  |
| **Fig. A91: Variation of reliability index of design models with service time for element 91** |
|  |
| **Fig. A92: Variation of reliability index of design models with service time for element 92** |
|  |
| **Fig. A93: Variation of reliability index of design models with service time for element 93** |
|  |
| **Fig. A94: Variation of reliability index of design models with service time for element 94** |
|  |
| **Fig. A95: Variation of reliability index of design models with service time for element 95** |
|  |
| **Fig. A96: Variation of reliability index of design models with service time for element 96** |
|  |
| **Fig. A97: Variation of reliability index of design models with service time for element 97** |
|  |
| **Fig. A98: Variation of reliability index of design models with service time for element 98** |
|  |
| **Fig. A99: Variation of reliability index of design models with service time for element 99** |
|  |
| **Fig. A100: Variation of reliability index of design models with service time for element 100** |
|  |
| **Fig. A101: Variation of reliability index of design models with service time for element 101** |
|  |
| **Fig. A102: Variation of reliability index of design models with service time for element 102** |
|  |
| **Fig. A103: Variation of reliability index of design models with service time for element 103** |
|  |
| **Fig. A104: Variation of reliability index of design models with service time for element 104** |
